# Could the Topping-Off Technique Be the Preventive Strategy against Adjacent Segment Disease after Pedicle Screw-Based Fusion in Lumbar Degenerative Diseases? A Systematic Review

**DOI:** 10.1155/2017/4385620

**Published:** 2017-02-22

**Authors:** Po-Hsin Chou, Hsi-Hsien Lin, Howard S. An, Kang-Ying Liu, Wei-Ren Su, Cheng-Li Lin

**Affiliations:** ^1^Department of Orthopedics and Traumatology, Taipei Veterans General Hospital, Taipei, Taiwan; ^2^School of Medicine, National Yang-Ming University, Taipei, Taiwan; ^3^Department of Orthopaedic Surgery, Rush University Medical Center, Chicago, IL, USA; ^4^Department of Orthopaedic Surgery, National Cheng Kung University Hospital, College of Medicine, National Cheung Kung University, Tainan, Taiwan

## Abstract

The “topping-off” technique is a new concept applying dynamic or less rigid fixation such as hybrid stabilization device (HSD) or interspinous process device (IPD) for the purpose of avoiding adjacent segment disease (ASD) proximal to the fusion construct. A systematic review of the literature was performed on the effect of topping-off techniques to prevent or decrease the occurrence of ASD after lumbar fusion surgery. We searched through major online databases, PubMed and MEDLINE, using key words related to “topping-off” technique. We reviewed the surgical results of “topping-off” techniques with either HSD or IPD, including the incidence of ASD at two proximal adjacent levels (index and supra-adjacent level) as compared to the fusion alone group. The results showed that the fusion alone group had statistically higher incidence of radiographic (52.6%) and symptomatic (11.6%) ASD at the index level as well as higher incidence (8.1%) of revision surgery. Besides, the HSD (10.5%) and fusion groups (24.7%) had statistically higher incidences of radiographic ASD at supra-adjacent level than the IPD (1%). The findings suggest that the “topping-off” technique may potentially decrease the occurrence of ASD at the proximal motion segments. However, higher quality prospective randomized trials are required prior to wide clinical application.

## 1. Introduction

Fusion surgery has been shown to improve functional outcomes in appropriately selected symptomatic patients with various degenerative lumbar disorders [1, 2]. However, adjacent segment disease (ASD) is still a significant problem following rigid spinal fixation [3, 4]. Fusion surgery aims to relieve symptom from degenerative or unstable motion segments. There is increase in range of motion and stress at the upper adjacent level after rigid fixation [3, 5], which is one of many factors, contributing to the development of ASD.

The incidence of radiographic ASD ranges from 5.2% to 100%, depending on patient population, follow-up duration, the imaging used for evaluation, and definition of ASD [4]. The symptomatic ASD ranged from 5.2% to 18.5% as reported by Park et al. [4]. Ghiselli et al. [3] reported the rate of symptomatic ASD following either decompression or fusion was predicted to be 16.5% at 5 years and 36.1% at 10 years.

Generally, symptomatic ASD in patients who failed in conservative treatment needs revision surgery to relieve symptoms. However, some studies reported relatively modest results in patients who received revision surgery for symptomatic ASD [6, 7]. Regarding the location of ASD, Aota et al. [8] demonstrated that ASD occurred in 24.6% of the cases proximal to lumbar fusion and 2.6% of the cases distal to fusion and a similar trend, reported by Etebar and Cahill [9]. It is important for surgeons to carefully evaluate the proximal adjacent disc above fusion levels before surgery in order to lower the occurrence of ASD.* The “topping-off” technique with either hybrid stabilization device (HSD) or interspinous process devices (IPD) might be one of the solutions.*

This “topping-off” technique refers to application of hybrid dynamic pedicle screw construct or interspinous process device above the fused segments. This technique provides a transitional zone between caudal rigid fused construct and cephalad mobile/unfused segments, which may decrease the incidence of ASD [10, 11]. The rationale of this technique is that the semirigid zone provides a gradual transition from the rigid to mobile segments to lessen stress concentration at the adjacent level. Khoueir et al. [12] classified posterior dynamic stabilization devices into three categories: (1) hybrid stabilization device with pedicle screw/rod construct such as DTO® and Dynesys (we defined it as HSD in this manuscript); (2) interspinous process devices (IPD) such as Wallis, X-STOP, DIAM, and Coflex; (3) total facet replacement system. Because of the lack of evidence in the literature on total facet implants, we focused on the former two devices of HSD and IPD in our literature reviews.

To our knowledge, systematic review investigating the “topping-off” technique with HSD or IPD to prevent ASD following lumbar fusion surgery has not been done. This manuscript reviews the surgical results of “topping-off” techniques and compares the incidence of ASD at proximal two adjacent levels among HSD, IDP, and fusion alone group.

## 2. Materials and Methods

We followed the methodological guidelines outlined by the Transparent Reporting of Systematic Reviews and Meta-Analyses (PRISMA) [19, 20] to conduct this systematic review.

### 2.1. Literature Search and Selection

A literature review of clinical studies published from January 2007 to December 2015 was conducted. The articles written in English were included. We completed a search into National Center for Biotechnology Information databases using PubMed/MEDLINE, with keywords and Boolean operators.* The search strategy for publications was of “Topping-off”, “hybrid stabilization”, “hybrid stabilization device”, “hybrid stabilisation”, “hybrid fixation”, and “interspinous process device” AND “fusion”, “lumbar spine”, “adjacent segment disease”, and “adjacent segment degeneration”. Editorials and commentaries from major neurosurgical and orthopaedic journals were also reviewed to gather further information on this topic. Furthermore, we searched and reviewed the relevant articles on the reference list for further information. We only *included* studies published in SCI (scientific citation index) journals.*

### 2.2. Methodological Quality Assessment

Full-text versions of all included articles were downloaded and assessed for potential bias by two independent reviewers (PC & CL). The National Heart Lung and Blood Institute (NIH) quality assessment tool for case series studies [21] was used to assess the methodological quality of the selected studies. This categorises studies as either good, fair, or poor. Encountering any disagreement, we made a consensus by discussion within the review team.

### 2.3. Article Selection and Data Extraction

We collected clinical trials studying the effect of hybrid stabilization or proximal IPD implantation to prevent ASD after lumbosacral fusion surgery. Many clinical studies were initially selected including prospective, retrospective studies or case series with or without comparison group (fusion alone). The problems adjacent to fusion levels or ASD were considered as primary outcomes. After reviewing the titles and abstracts of collected studies, we then determined if the content of the studies was suitable for retrieval. The studies in which the average patient follow-up time was less than 24 months or the number of patients was less than 20 were not considered.

Two authors independently extracted data from the articles. We contacted the authors of the studies for the uncertain details. The following data were extracted: (1) participant demographics; (2) indication for surgeries; (3) adjacent segment degeneration; (4) radiographical and clinical outcomes; (5) implant-related complications and other outcomes. Details of ASD following fusion surgery and required revision surgery were further analyzed among the three groups.* Only ASD that were specifically stated as having occurred or not having occurred in the articles were used in the analysis. ASD were not assumed to be absent just because they were not discussed* (Table 4).

### 2.4. Statistical Analysis

For statistical analysis, quantitative data are described by the mean, range, and standard deviation if available; qualitative data are described as counts and percentages. We used chi-square test with the Yates continuity correction to evaluate the incidence or proportion in the comparative groups in the parameters. A *p* value of < 0.05 indicated statistical significance. All statistical computation has been performed with the SPSS for Windows statistical package (version 21.0, Chicago, Illinois).

## 3. Results

### 3.1. Identified Trials

**Figure 1:**
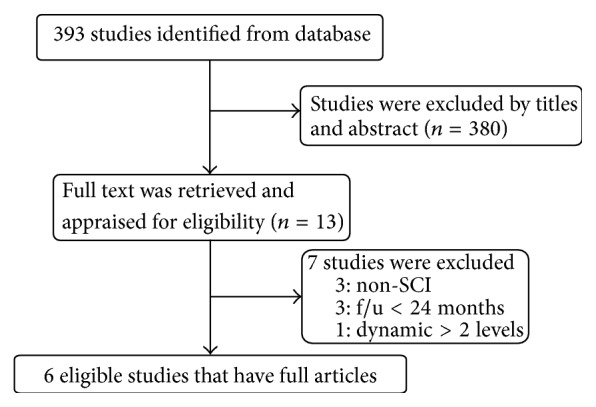
The flow chart for manuscript selection.

A flow chart describing the procedure of study selection is shown in Figure 1. The search yielded 393 articles of prospective or retrospective case series. No additional studies were found manually. All studies had abstracts screened and assessed for eligibility. Thirteen full-text articles were retrieved and appraised for eligibility. Eventually 366 patients from 6 articles, 2 prospective [12, 14] and 4 retrospective [13, 15–17], were included in our systematic review. The methodological quality as measured by the NIH quality assessment tool was high with all studies assessed as good. The level of evidence for these selected articles was also analyzed (Table 1).

### 3.2. Study Characteristics and Outcomes

The relevant characteristics for each included study are summarized in Table 1. Regarding the level of evidence, there were two papers of level II [14, 18], three papers of level III [15, 16, 22], and one paper of level IV [13]. Every particular indication for “topping-off” surgery was reported in all studies. Some degree of adjacent disc degeneration was the main reason for dynamic stabilization above fusion construct in most (5/6) studies. Location for topping-off stabilization was illustrated in 5 studies, located from L1/2 to L4/5. The methodology for evaluating radiographic and clinical results was not consistent in all studies. The evaluation tools and results in each study are summarized in Tables 2 and 3, respectively, for radiographic parameters and functional outcomes. The radiographic evaluation tools used in these studies are disc height, foraminal height, and UCLA grade obtained from plain radiography and Pfirrmann's classification and Modic grade obtained from MRI images. It is difficult to compare the radiographic results among these studies because of the inconsistency of evaluation tools (Table 2). The clinical outcome was evaluated with visual analogue score (VAS) for back or leg, Oswestry Disability Index (ODI), and short form (36) health survey (SF-36). Ultimately, all studies revealed that the clinical outcomes improved significantly postoperatively (Table 3).

### 3.3. Adjacent Segment Disease

**Figure 2:**
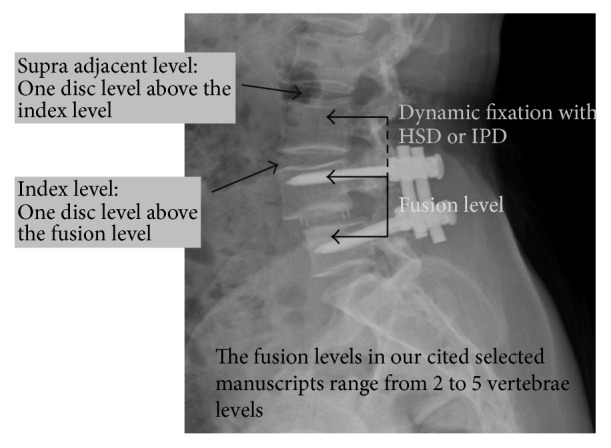
The definitions of “index level” and “supra-adjacent level” in our manuscript. “Index level” represents the disc level just above the fusion construct. “Supra-adjacent level” represents one disc level above the index level. The fusion levels in selected manuscripts ranged from 2 to 5 vertebrae levels, which were not, respectively, presented herein.

The demographic data and results of ASD for the topping-off techniques and fusion alone group were listed in Table 4. There were 95 patients in HSD group, 98 patients in IPD group, and 173 patients in fusion alone group with a mean age of 62.7, 64.9, and 60.5, respectively. The number of fused vertebrae was 2 in HSD group, 3.4 in IPD group, and 2.5 in fusion alone group. The mean follow-up time was 42.8, 47.2, and 50.4 months in each group. The details of adjacent segment disease for topping-off techniques and fusion alone group are shown in Tables 5 and 6, respectively. The definitions of “index level” and “supra-adjacent level” were illustrated in Figure 2.

#### 3.3.1. ASD at the Index Level

The index level was defined as the level of HSD or IPD or the adjacent level above fusion. The difference in the incidence of radiographic or symptomatic ASD at the index level was statistically significant among the three groups. The fusion group presented statistically higher percentage of symptomatic ASD (11.6% or probably higher as some papers defined ASD requiring revision surgery for symptomatic ASD) and radiographic ASD (52.6%) as well as revision surgery for ASD (8.1%) as compared to “topping-off” groups (*p* = 0.003, *p* < 0.001, and *p* = 0.008 resp.).

#### 3.3.2. ASD at Supra-Adjacent Level

The supra-adjacent level was defined as the level above index level. Interestingly, the HSD (10.5%, 7 out of 95 patients) and fusion groups (24.7%, 20 out of 81 patients) had higher incidences of radiographic ASD at supra-adjacent level than in the IPD (1%, 1 out of 98 patients) (*p* < 0.001). The fusion alone group still had a higher incidence of ASD at supra-adjacent level as compared to HSD (*p* < 0.05).

### 3.4. Implants-Related Complications in HSD or IPD

No implant-related complication was reported in all IPD group. Regarding the HSD group, a patient needed revision surgery after 26 months because of a clinically symptomatic dislocation of the Dynesys screws. This patient was excluded from further analysis because dynamic stabilization was removed during revision surgery.

## 4. Discussion

Accelerated degeneration at adjacent segments above or below lumbar spinal fusion site has been a significant problem in clinical practice. In this review, we focused on the cephalad “topping-off” techniques either HSD or IPD and compared with the fusion alone groups, as these newer techniques are controversial. Our review revealed the potential of these “topping-off” techniques in decreasing the incidence of ASD after fusion surgery.

### 4.1. The Mechanism of ASD

While rigid fixation improves the fusion rate and functional outcomes [1, 2], many studies have reported the increased prevalence of adjacent motion segment degeneration following lumbar fusion [3, 4, 7]. Although clinical studies investigated risks factors predisposed in the progression of ASD [5, 8, 9, 23–30], the exact pathogenesis of ASD remains uncertain. Biomechanical and clinical studies have suggested the compensatory loading transfer [31] and increased range of motion [3, 5] at upper adjacent level after rigid fixation. Regarding the intradiscal pressure (IDP) at proximal adjacent disc (PAD) following rigid fixation, Cunningham et al. [32] reported an increase of IDP up to 45% on axial compression and anterior flexion loading motion in comparison to normal disc. Weinhoffer et al. [33] also reported a significant increase of IDP at PAD following instrumentation in a simulated fusion model. The authors mentioned increased IDP may alter the metabolic status and further play an important role in the pathogenesis of ASD. However, there are several clinical studies suggesting that ASD is part of a normal degenerative process rather the altered biomechanical stress on the adjacent disc [34, 35].

### 4.2. The Risk Factors for ASD

There are many papers on the risks factors for ASD. These risk factors include patient's age [8, 9, 24], postmenopausal status [9], sagittal mal-alignment [5, 25, 26], multiple level fusion [23, 28, 29], posterior interbody fusion [24], iatrogenic injury to the facets of the adjacent segment [8, 30], and preexisting disc degeneration [36]. There are other papers in the literature supporting or contradicting these risk factors [4]. Kumar et al. [37] reported that gender, different types of fusion (posterior fusion versus combined posterolateral and posterior interbody fusion), and fusion level (fusions extending down to the sacrum versus fusions stopped at short of the sacrum) are not risk factors for ASD. In addition, Rahm and Hall [24] reported a negative correlation between sagittal alignment and incidence of ASD. The inconsistent conclusions are as a result of retrospective selection bias, limited follow-up time, or different methodology evaluating ASD. The progression of ASD following lumbar spine fusion is obviously multifactorial, and further research can help identify and quantify the contributing risk factors for ASD.

### 4.3. Intervals from Fusion Surgery to Revision Surgery for Symptomatic ASD

Based on Lee et al. [22], Kumar et al. [5], and Aota et al. [8], the mean interval from fusion to revision surgery for ASD is approximately 51 months, ranging from 41.3 to 62.4 months. We excluded studies with limited follow-up time less than 24 months and the occurrence of ASD is greater with longer follow-up.

### 4.4. Biomechanical Characteristics in Dynamic Devices on Spine Range of Motion (ROM) and Intradiscal Pressure (IDP)

Schmoelz et al. [38] reported Dynesys does not change IDP at proximal adjacent disc after fixation under moment-controlled mode, while Cabello et al. [39] reported Dynesys decreases 50% of the IDP at instrumented level and increases 10% of the IDP at supra-adjacent level under load-controlled mode. Different controlled modes in biomechanical testing may explain these diverse results [40]. Moreover, Schmoelz et al. [38] reported Dynesys is more flexible than rigid fixation, but spine ROM was still limited.

Lafage et al. [41] reported that the Wallis decreases the disc stress and ROM and increases the spinous process loading at instrumented level. Bellini et al. [31] reported that DIAM in vitro decreases ROM and IDP at instrumented level. The Wallis and DIAM both decrease but preserve some degree of ROM [31, 41], which can decrease the stresses at the adjacent level.

### 4.5. Rationale of “Topping-Off” Technique and Clinical Application

The “topping-off” technique provides a transitional zone between caudal rigid fused segment and cephalad mobile unfused spines, which may decrease the incidence of ASD [10, 11]. Based on posterior dynamic stabilization system reported by Khoueir et al. [12], the Dynesys construct belongs to hybrid stabilization device; Wallis and DIAM belong to posterior interspinous device. Similar biomechanical characteristics include decreased IDP and limited [31, 38, 39, 41, 42] but still preserve some ROM at HSP/IPD instrumented level.

Based on this systematic review, the incidences of radiographic ASD at index level were 12.6%, 10.2%, and 52.6% in HSD, IPD, and fusion alone, respectively. With the “topping-off” technique, the incidence of ASD seems to decrease significantly at mid-term follow-up. These devices might possibly alleviate the degenerative progression above the fusion level. Regarding the incidence of radiographic ASD at supra-adjacent level, there were 1%, 10.5%, and 24.7% in IPD, HSD, and fusion alone, respectively. The IPD has the best result in delayed progression of ASD at supra-adjacent level. From the biomechanical view, we assumed that the HSD was more rigid than IPD but less rigid than the instrumented fusion, which may be one of the explanations for the results. Another possible reason for higher incidence of ASD at supra-adjacent level in HSD comparing to IPD is that iatrogenic facet joints surface might jeopardize when placing proximal pedicle screws [8, 43]. More in vitro biomechanical and high-quality prospective randomized studies are needed for further clarification on the issue.

### 4.6. Implants- (HSD or IPD) Related Complications

The incidence of broken pedicle screws in treatment of degenerative lumbar disease ranged from 2.2% to 12.4% [44–46] based on either total pedicle screws or patient numbers. In our results, 2 broken dislodged dynamic screws in 1 patients (0.98%, 1 out of 102 patients) in HSD group were observed, which was much lower than traditional pedicle screws fixation. This result could be different if more studies were to be analyzed or if follow-up was longer.

After Wallis being implanted, there is a change in the stress distribution of the spine, especially the spinous process [41]. Moreover, application of the tension band construct significantly increases the stress of the contact surface between the spinous process and the implant. Significant bone resorption was observed in more than 50% of the patients with Wallis implantation as reported by Wang et al. [47] and by Miller et al. [48]. The possible reasons to explain spinous process fracture or resorption are as follows: (1) the downward conduction of stress in the lumbar spine at greatest force at L5 spinous process [49]; (2) continuous motion at implanted level. Nevertheless, neither bone resorption nor spinous fracture was observed in this review.

### 4.7. Can Preoperative Disc Degeneration Affect the Incidence of ASD after Fusion?

*Park et al. [4] reported that the preoperative condition of adjacent disc for further implication in ASD following fusion is still elusive.* Ghiselli et al. [3] reported the correlation between ASD and preoperative disc degeneration status at the time of surgery is not significant in 215 patients based on UCLA disc degeneration grading with mean 7-year follow-up. Nakai et al. [36] and Liang et al. [50] reported the preoperative disc degeneration correlates with the progression of ASD at adjacent fusion level based on the disc height and pre-MRI Pfirrmann's grading, respectively. All these studies did not perform the postoperative MRI image to evaluate the disc degenerative status as final follow-up.

Preoperative disc degeneration with Pfirrmann grade ≧ III [50, 51] has a higher chance of developing symptomatic ASD. Regarding relative risks (RR) for developing ASD after fusion surgery, Ghiselli et al. [3] reported L4-5 poses a high risk, T12-L1, L1-2, and L3-4 have the intermediate risks, and L2-3 has the lower relative risks. Liang et al. [50] reported disc bulge in preoperative CT examination may serve as reasonable prediction for symptomatic ASD. Sénégas [52] reported that Wallis can be used for disc degeneration of Pfirrmann's classification grades II, III, and IV above the fusion level.

Taken together, surgeons should be more aware of preoperative adjacent disc condition. The reasonable indications for “topping-off technique” might be (1) Pfirrmann Gr. ≧ III, (2) budged disc, and (3) high risk (L4-5) disc level and relative intermediate risk disc levels (T12-L1, L1-2, and L3-4). However, we found inconclusive surgical indications for topping-off fixation in this systematic review. We still need more evidence to support this conclusion by prospective randomized controlled study. Nevertheless, we suggest surgeons to pay more attention to the preoperative adjacent disc degenerative status, correlating between radiographic findings and patients' symptoms. The patient should be informed on the controversial nature and unpredictable outcomes when inserting these devices. More importantly, surgeons could improve their surgical techniques, such as maintaining the lordosis at the instrumented levels [5, 25, 26], no violation of the proximal adjacent facet joints [53, 54] when placing pedicle screws at the most upper levels, and no* excessive distraction of disc for interbody fusion [55]. *These techniques will likely lessen the development of ASD.

### 4.8. Limitations

Several major drawbacks or limitations were found in this systematic review. First, a small number of enrolled patients and short follow-up time do not lead to a definitive conclusion. Second, there could be selection bias. Third, the criteria of radiographic parameters for ASD were not consistent in these cited studies. We suggest using MRI images combined with flexion-extension radiography to diagnose ASD if feasible. Based on our literature review, the application of “topping-off” technique with HSD or IPD above fusion to avoid ASD still lacks good evidence, and therefore prospective randomized clinical trials should be conducted to further elucidate the role of topping-off techniques.

## 5. Conclusion

Although the evidence is weak, the “topping-off” technique with HSD or IPD might decrease the incidence of proximal ASD both radiographically and symptomatically as compared to the fusion group. At the index level, the effects of HSD or IPD for decreasing ASD were similar. At supra-adjacent level, IPD seems to have the better effect of avoiding ASD. In conclusion, the “topping-off” technique might be considered as a possible solution for postfusion ASD, but further research is needed prior to wide application. The patient selection and choices of stabilizing implants should be assessed with more level I clinical studies. Based on our literatures review, the preventive strategy of ASD with application of “topping-off” technique above fusion is still elusive, and prospective randomized trials with higher quality are still required for further elucidating the effect of topping-off technique for prevention of ASD.

## Figures and Tables

**Table 1:** Clinical reported hybrid stabilization device and interspinous process device in the lumbar degenerative surgery.

Authors, years	Implant type	Study (level of evidence)	Patients numbers	Mean F/u (months)	Min. F/u (months)	Max. F/u (months)	Indications for index level instrumentation	Exclusion criteria for the study	Fused vertebrae numbers	Level for dynamic device	Reported radiographic outcomes	Reported clinical outcomes
*Hybrid stabilization device (HSD)*												
Formica et al. [13], 2015	CD HORIZON BalanC™ System (Medtronic Minnesota, USA)	Retrospective series cases (IV)	38	24	24	24	Pfirrmann I to III	(1) Idiopathic or deg. scoliosis (2) Gr. 3-4 listhesis (3) Failed back syndrome (4) Sagittal imbalance (LL < PI-5°) (5) BMI > 35 (6) Clinical contraindication to surgery^*∗*^	2	L23 L34 L45	X-ray (DH/VBH) MRI (Pfirrmann)	VAS (back, leg) ODI

Putzier et al. [14], 2010	Allo Spine™ Dynesys Transition System (Zimmer, Winterthur, Switzerland)	Prospective randomized, nonblind comparative (II)	22	76.4	60	91	Asymptomatic but radiographic DD (Modic grade I)	(1) Symptomatic proved by discography (2) Positive disc analgesia (3) Severe facet joints arthropathy (4) Spine deformity (5) Destructive process (6) Previous surgery	2	L34 L45	MRI (Modic)	VAS (back) ODI

Imagama et al. [15], 2009	Natural Neutral Concept Rod (Howa Co. Ltd.)	Retrospective comparative (III)	35	42	NA	NA	No instability	(1) ≧4 mm listhesis at L3 (2) Severe DD at L34 (Pfirrmann's V) (3) Deg. scoliosis ≧ 10°(4) Severe instability	2	L34	X-ray (middle DH) MRI (Pfirrmann)	JOA score

*Interspinous process device (IPD) *												
Lu et al. [16], 2015	DIAM (Device for Intervertebral Assisted Motion) (Medtronic Sofamor Danek)	Retrospective (III)	49	41.2	24	48	(1) DH ≦ 50% with/without segmental F/E mobility (2) No segmental F/E mobility and the status of the discs was suitable	NA	4	L12 L23 L34	MRI (Pfirrmann)	VAS (back, leg) ODI

Lee et al. [17], 2013	DIAM (Device for Intervertebral Assisted Motion) (Medtronic Sofamor Danek)	Retrospective (III)	25	46.8	24	97	(1) Pfirrmann Gr. II-III (2) MRI: facet deg. or effusion (3) Mild to moderate spinal or foraminal stenosis	NA	2	L23 L34 L45	X-ray (FH)	VAS (back, leg) ODI

Korovessis et al. [18], 2009	Wallis	Prospective controlled (II)	24	60	NA	NA	(1) UCLA Gr. I or II (no listhesis or lytic lesion) (2) Degenerative (listhesis spinal stenosis, loss of segmental lordosis) (3) 2 to 4 vertebral fusions	(1) Severe osteoporosis (2) Loss lumbar lordosis (3) Previous lumbar surgery (fracture) (4) Ankylosis (5) UCLA > II in index level (6) Spinous process insufficiency	3.5	NA	X-ray (ant./post. DH) (UCLA)	VAS (back) ODI SF-36

NA: not available, min.: minimum, max.: maximum, f/u: follow-up, PSs: pedicle screws, FH: foraminal height, ant.: anterior, post.: posterior, deg.: degenerative, F/E: flexion/extension, ASD: adjacent segment disease, ODI: Oswestry Disability Index, VAS: visual analogue scale, JOA: Japanese Orthopaedic Association, DH: disc height, and VBH: vertebral body height.

^*∗*^Other clinical contraindications were including (1) long-term medication of steroid or NSAID (chronic pain Gerbershagen Gr. ≧ 2); (2) liver or kidney diseases; (3) malignant tumor; (4) pregnancy; (5) chronic nicotine, alcohol, or drug abuse.

**Table 2:** Reported radiographic parameters in the hybrid stabilization device and interspinous process device groups.

Authors, years	Pre-op DD grading		X-ray DH at dynamic level			MRI grading at dynamic level		X-ray disc height at one level above			Global lumbar lordosis			Instrumented lumbar lordosis			Segmental motion at index level			Segmental motion at supra-adjacent level		
	X-ray	MRI	Pre-op	Post-op	Final	Pre-op	Final	Pre-op	Post-op	Final	Pre-op	Post-op	Final	Pre-op	Post-op	Final	Pre-op	Post-op	Final	Pre-op	Post-op	Final
Formica et al. [13], 2015	DH/VBH	Pfirrmann				I to III		0.278 (0.032)	0.282 (0.027)	0.269 (0.041)				49.56 (7.38)	56.57 (7.34)	56.9 (7.21)						

Putzier et al. [14], 2010		Modic				I																

Imagama et al. [15], 2009	Middle third	Pfirrmann	10 (2.1)		9.8 (2.3)	III to V	Progressed one grade in 4 pts	9.9 (1.6)		9.4 (2.2)	42 (15)		45 (14)				7.9 (4.3)		4.6 (3.8)	6.7 (2.7)		7.2 (3.1)

Lu et al. [16], 2015		Pfirrmann				Mean Gr. 2.9 I: 6, II: 16 III: 16, IV: 14					40. 6	36.2					35/49 (71%) maintained pre-op F/E motion					

Lee et al. [17], 2013	Foraminal		Foraminal height														5.9	6.5	4.9			
	height		21.3	24.4	21.6													(1 yr)				

Korovessis et al. [18], 2009	DH (ant.) DH (post.) UCLA^+^	11.9 (ant. DH)	13.8 (1 yr)	12																		
		7.7 (post. DH)	8.2 (post-op)	7.9							39.7	40.9 (1 yr)	42.8				5	4.8 (1 yr)	4.5			
		(Estimated from figures)									(Estimated from figures)						(Estimated from figures)					

Blank in the each column meant not mentioned by the authors. UCLA^+^: University of California at Los Angeles (UCLA) grading scale. The values in the parentheses were standard deviation, deg.: degenerative, pre-op: preoperative, post-op: postoperative, and F/E: flexion/extension.

DD: disc degeneration, ant.: anterior, post.: posterior, DH: disc height, VBH: vertebral body height, and pt: patients.

**Table 3:** Reported functional outcomes in the hybrid stabilization device and interspinous process device groups.

Authors, years	Patients	Mean	Visual analogue scale (back)			Visual analogue scale (leg)			Oswestry Disability Index			SF-36		
		f/u	Pre-op	Post-op	Final	Pre-op	Post-op	Final	Pre-op	Post-op	Final	Pre-op	Post-op	Final
Formica et al. [13], 2015	38	24	7.87 (1.39)	1.98 (1.04)	0.42 (0.53)	4.77 (1.98)	1.87 (1.55)	0.37 (0.9)	62.18 (13.1)		18.11 (4.78)			

Putzier et al. [14], 2010	22	76.4	8	4	4				70	30 (1 yr)	35			
			(Estimated from figures)						(Estimated from figures)					

Imagama et al. [15], 2009	35	42							JOA					
									11.8 (6.2)	25.2 (3.7)				
									

Lu et al. [16], 2015	49	41.2	7.1 (1.4)	1.3 (2 yrs) (2.3)	1.5 (2.4)	7.2 (1.3)	1.4 (2 yrs) (2.5)	1.4 (2.3)	27.7 (3.8)	14.6 (2 yrs) (3.4)	14.1 (3.9)			
		

Lee et al. [17], 2013	25	46.8	7.2	4.3 (1 yr)	3.9	6.9	3.8 (1 yr)	3.7 (2 yr)	26.1	17.4 (1 yr)	16.3 (2 yrs)			

Korovessis et al. [18], 2009	24	60	7.2 (2.1)	3 (2)					34	8	9	11	61	59
									(Estimated from figures)					

The values were presented as mean, the values in the parentheses were standard deviation, and blank in the each columns meant not mentioned in the manuscript.

JOA: Japanese Orthopaedic Association Score; SF-36: short form-36 questionnaires.

**Table 4:** Data in the hybrid stabilization device, interspinous process device, and fusion groups.

	Hybrid stabilization device	Interspinous process device	Fusion	*p* value
*Numbers of patients*	95	98	173	NA
*Age (y/o)* ^*+*^	62.7	64.9	60.5	NA
*Male/female*	NA	NA	NA	NA
*Numbers of fused vertebrae*	2	3.4	2.45	NA
*Follow-up (months)*	42.8	47.2	50.4	NA
*Adjacent segment disease (ASD)*				
Symptomatic ASD at index level	0	5 (5.1%)	20^++^ (11.6%)	**0.003**
Radiographic ASD at index level	12 (12.6%)	10 (10.2%)	91 (52.6%)	**<0.001**
Symptomatic ASD at supra-adjacent level	0	0	0	**—**
Radiographic ASD at supra-adjacent level	7 (10.5%)	1 (1%)	20/81^*∗*^ (24.7%)	**<0.001**
Revision surgery for ASD	0	3 (3%)	14 (8.1%)	**0.008**

The bold numbers in the *p* values indicated statistical significance. NA indicates not available.

^+^The authors did not exclude those who lost follow-up in the demographic results. The mean age was just estimated.

^++^Two papers only mentioned numbers of revision surgeries for symptomatic ASD but did not mention numbers of symptomatic ASD. (The result might be underestimated.)

^*∗*^Only 3 cited manuscripts reported their results.

**Table 5:** Adjacent segment disease and implant-related complications in hybrid stabilization device and interspinous process device.

Authors, years	Symptomatic ASD at index level or supra-adjacent level	Radiographic ASD at index level or supra-adjacent level	Criteria for radiographic ASD	Dynamic implant- related complications	Reasons for revision	
					Symptomatic ASD	Implant failure
Formica et al. [13], 2015	0 pts: index level 0 pts: supra-adjacent level	0 pts: index level 0 pts: supra-adjacent level	NA	No	No	No

Putzier et al. [14], 2010	0 pts: index level 0 pts: supra-adjacent level	2 pts.: index level 1: fusion, 1: instability 2 pts: supra-adjacent level 2: progressive DD	(1) Fusion (2) Disc degeneration Modic > 1 (3) Facet arthritis, Fujiwara > Gr. 1 (4) <25% pre-op disc height (5) Instability signs such as traction spurs	1 pt: dynamic PSs dislocation at 26 months Removed implant (the pt was excluded in the study)	No	1^+^

Imagama et al. [15], 2009	0 pts: index level 0 pts: supra-adjacent level	10 pts: index level (7 pts by MRI) (3 pts diagnosed by X-ray^*∗*^) 5 pts: supra-adjacent level (2 pts by MRI) (3 pts diagnosed by X-ray^*∗*^)	(1) DD (Pfirrmann) progression ≧1 grade (2) Spinal stenosis progression ≧ 1 grade	No	No	No

Lu et al. [16], 2015	3 pts: index level 0 pts: supra-adjacent level	3 pts: index level 0 pts: supra-adjacent level	(1) Anterolisthesis (2) Retrolisthesis due to hypermobility on flexion/extension (3) Loss of disc height and sclerosis along endplate (DD)	Spinous process fr.: no Implant failure: no	1	No

Lee et al. [17], 2013	2 pts: index level NA: supra-adjacent level	6 pts: index level NA: supra-adjacent level	(1) Collapsed disk space (Pfirrmann Gr. V) (2) Spondylolisthesis (translation ≧3 mm) (3) Proximal junctional kyphosis Cobb angle ≧10°(4) Compression fr. at adjacent segments	Spinous process fr.: no Implant failure: NA	2	NA

Korovessis et al. [18], 2009	0 pts: index level 0 pt: supra-adjacent level	1 pt: index level 1 pt: supra-adjacent level	(1) Listhesis (2) Disc collapse (3) ↑segmental range of motion (ROM) (4) >grade II of modified UCLA grade	Spinous process fr.: no Implant failure: NA	0	NA

NA: not available. Numbers: occurrence of patients numbers. Pre-op: preoperative.

^*∗*^Radiographic ASD defined as one of the following criteria in X-ray: (1) disc height decrease ≦ 50%; (2) listhesis ≧ 3 mm (neutral position); (3) disc angle decrease (at flexion) ≧5°.

ASD: adjacent segment disease, pts: patients, PSs: pedicle screws, DD: disc degeneration, DIAM: Device for Intervertebral Assisted Motion, f/u: follow-up, and fr.: fracture. ^+^1 pt. suffered clinically symptomatic dislocation of dynamic pedicle screws and needed revision surgery of implant removal at 26 months f/u and was excluded in the study.

**Table 6:** Adjacent segment disease in the fusion alone groups in these cited manuscripts.

	Patients numbers	Mean age (Y/O)	Pre-op status at index level^*∗*^	Radiographic ASD at index level	Symptomatic ASD at index level	Revision surgery for ASD	Radiographic ASD at supra-adjacent level
Formica et al. [13], 2015	No comparative fusion group in the manuscript						

Putzier et al. [14], 2010	25	44.6	(1) Asymptomatic bur radiographic DD (Modic grade I)	6	1	1	0

Imagama et al. [15], 2009	35	64	(1) No instability (2) Pfirrmann Gr. II–IV (3) None, mild, or moderate spinal stenosis	35	No	No	14

Lu et al. [16], 2015	42	59	(1) DH ≦ 50% with/without segmental F/E mobility (2) No segmental F/E mobility and the status of disc was suitable	20	9	3	NA

Lee et al. [17], 2013	50	65.9	(1) Pfirrmann Gr. II-III (2) MRI: facet degeneration or effusion (3) Mild to moderate spinal or foraminal stenosis	24	7^+^	7	NA

Korovessis et al. [18], 2009	21	64	(1) UCLA Gr. I or II (no listhesis or lytic lesion) (2) Degenerative (listhesis, spinal stenosis, loss of segmental lordosis) (3) 2 to 4 vertebral fusions	6	3^+^	3	6

DH: disc height; F/E: flexion/extension. ^+^The authors only mentioned numbers of revision surgeries for symptomatic ASD but did not mention numbers of symptomatic ASD. The numbers of symptomatic ASD might be underestimated.
